# A Mini-Review on the Thermal Fatigue Properties of Copper Materials Applied at the Front-End of Synchrotron Radiation Facilities

**DOI:** 10.3390/e25050714

**Published:** 2023-04-26

**Authors:** Yunfei Sun, Tong Li, Lan Lan, Jiahua Chen, Wanqian Zhu, Song Xue, Limin Jin

**Affiliations:** 1 Shanghai Institute of Applied Physics, Chinese Academy of Sciences, Shanghai 201800, China; 2 University of Chinese Academy of Sciences, Beijing 100049, China; 3 Shanghai Synchrotron Radiation Facility, Shanghai Advanced Research Institute, Chinese Academy of Sciences, Shanghai 201204, China

**Keywords:** thermal fatigue, crack length, plastic strain, synchrotron radiation, front-end

## Abstract

Oxygen-free high-conductivity copper (OFHC), chromium-zirconium copper (CuCrZr), and Glidcop^®^ AL-15 are widely used in the high heat load absorber elements at the front end of synchrotron radiation facilities. It is necessary to choose the most suitable material according to the actual engineering conditions (such as the specific heat load, material performance, and costs). In the long-term service period, the absorber elements have to bear hundreds or kilowatts of high heat load and its “load-unload” cyclic loading mode. Therefore, the thermal fatigue and thermal creep properties of the materials are critical and have been extensively studied. In this paper, based on the published pieces of the literature, the thermal fatigue theory, experimental principles, methods, test standards, test types of equipment, and key indicators of the thermal fatigue performance of typical copper metal materials used in the front end of synchrotrons radiation Facilities are reviewed, as well as the relevant studies carried out by the well-known synchrotron radiation institutions. In particular, the fatigue failure criteria for these materials and some effective methods for improving the thermal fatigue resistance performance of the high-heat load components are also presented.

## 1. Introduction

The front end between the electron storage ring and the beamline plays a very important role in a synchrotron radiation facility. When the synchrotron beam is extracted from the storage ring, hundreds or thousands of kilowatts of heat are generated, of which only a small part is introduced into the beamline, and the experimental station, most of the heat must be absorbed by the front end. The core components of the front end are various types of photon absorbers (photon shutters, fixed maskers, etc.). The general functions of the front end are defining the beam size, absorbing the high heat load, vacuum protection, beam position measurement, etc. Among them, defining the beam size and absorbing the high heat load are the main functions [1,2,3].

Currently, the energy of synchrotron radiation is constantly increasing to meet the ever-changing demands of scientific research. It leads to a series of problems, such as the inability of absorber elements to withstand the high heat load, which include thermal creep, thermal fatigue damage, etc. Although there are a series of methods to deal with the above problems, such as using the grazing incidence structure for the beam irradiation surface to reduce the thermal power density, improving the convective heat transfer coefficient at the cooling tube inner wall [4], and using the high-performance copper (such as Glidcop^®^AL-15) [5,6]. However, the thermal fatigue life is not directly considered when designing the components. In practice, the temperature gradient between the high-temperature region and low-temperature region of the beam irradiation surface is relatively large, resulting in great thermal stress. During the long-term service period, the beamlines are always in the cycle of running and stopping. As a result, all components in the front end are subject to rapid cooling and heating many times, and their mechanical properties are liable to decline due to thermal fatigue. Therefore, the long-term accumulation of this issue may make the thermal stress exceed the thermal fatigue yield limit of the applied material and cause plastic deformation. Thus, fatigue cracks will be generated, and finally, failure will occur due to the crack growth [7]. Furthermore, thermal fatigue damage may occur at any time, and it is difficult to be detected in time. Therefore, it is especially important to avoid thermal fatigue damage at synchrotron radiation facilities.

In addition, thermal creep is another important factor. It is caused by high temperature and high pressure and can also lead to the failure of components. The thermal creep of copper metal is the phenomenon that plastic deformation occurs slowly, undergoing the long-term constant high heat load [8,9]. At low-temperature conditions, the effect of thermal creep can be ignored. However, its impact cannot be ignored at the synchrotron front end. Therefore, thermal fatigue and thermal creep are two important issues that must be considered for the high heat load components.

In summary, the thermal fatigue and creep of metallic materials need to be taken into account when upgrading or optimizing the performance design of the high heat load components at the front end of synchrotron radiation facilities, which will positively contribute to safety, operational stability, and economic rationality.

This paper reviews the thermal fatigue theory, experimental principles, methods, test standards, and types of equipment, as well as key indicators and fatigue failure criterion of copper metal materials used for the high heat load elements in the front end of synchrotron radiation facilities and provides an overview of the relevant studies of the representative synchrotron instruments to provide useful guidance for the optimal design of thermal fatigue-resistant components.

## 2. Thermal Fatigue Theory of Metals

### 2.1. Thermal Stress and Thermal Strain

When thermal deformation caused by temperature change is restrained, stress will be generated inside the material, which is called thermal stress, and the strain is called thermal strain.

**Figure 1:**
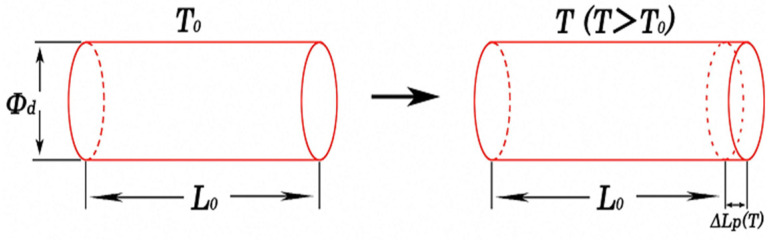
Schematic diagram of the cylindrical body subjected to thermal expansion.

As shown in Figure 1, for a cylinder with diameter d, its length at room temperature *T*_0_ is *L*_0_ (mm), and the coefficient of linear expansion is α (/°C); when the temperature changes to *T* (°C), the length changes by *∆L_f_* (*T*), which can be expressed by Equation (1) [10]:

(1) $$\Delta L_{f}\left(T\right)=\alpha L_{0}(T-T_{0})$$

Suppose this cylinder is completely restrained at room temperature *T*_0_ by a rigid frame at both ends, and the specimen is not bent and buckled when the temperature changes. The strain *ε°*(*T*) can be expressed in the form of Equation (2):

(2) $$\varepsilon {}^{\circ}\left(T\right)=-\Delta L_{f}\left(T\right)/L_{0}=-\alpha (T-T_{0})$$

The negative sign in Equation (2) indicates that the strain is opposite to the temperature change phase. The thermal stress *F°* (MPa) can be obtained by Hooke’s law:

(3) $$\;F{}^{\circ}=E\varepsilon {}^{\circ}\left(T\right)=-E\alpha (T-T_{0})$$

where $\Delta L_{f}\left(T\right)$ is the length change (m) in the unconstrained state when the temperature goes from *T*_0_ (°C) to *T* (°C), $L_{0}$ is the original length (m) at temperature *T*_0_ (°C), α is the coefficient of linear expansion (°C), F° is the thermal stress (MPa), and E is the Elastic modulus (MPa).

### 2.2. Thermal Fatigue Strength

For thermal fatigue, the number of cycles (*N_f_*) required to reach the ultimate breakage or failure is named the thermal fatigue strength.

Back in the early 1950s, the famous Manson–Coffin formula explained the relationship between strain range and thermal fatigue strength.

(4) $$\varepsilon_{P}N_{f}{}^{\alpha }=C$$

where *ε_p_* is the plastic strain range, *N_f_* is the number of cycles to failure, and α and *C* are the material constants related to the test conditions.

The Manson–Coffin formula has been widely used for the prediction of finite life of constant amplitude fatigue, and the formula has been extended to short fatigue cracking, variable amplitude fatigue, etc. The modified Manson–Coffin formula has been applied to various aspects, such as multi-axial fatigue and thermal stress fatigue.

The physical significance of this formula is that the mechanical properties can be used to predict fatigue life. However, the material constants, such as coefficients and exponents, in this formula need to be determined by special experiments or calculated using empirical relationships with mechanical properties, and more importantly, the quantitative relationship between fatigue life and crack length cannot be determined [11,12,13].

In addition, unlike the mechanical fatigue test, the thermal fatigue test of metals is generally a qualitative test in which the specimen is subjected to only cyclic temperature, resulting in fatigue damage under the repeated action of cyclic thermal stress. Therefore, the ability of metallic material to resist thermal fatigue damage is named thermal fatigue resistance, which is one of the important indicators for evaluating the thermal fatigue performance of metal materials [14]. It is expressed as the crack length for a specified number of cycles or the number of cycles for a specified crack length. The tests have the advantages of a relatively short test time, low cost and reliable test data.

## 3. Thermal Fatigue-Related Studies in the Typical Synchrotron Institutions

For the thermal fatigue issue of the high heat load copper components applied for the front end of synchrotron radiation facilities, some well-known synchrotron institutes, such as SPring-8 (Japan) and APS (USA), have conducted a series of studies and made significant achievements.

### 3.1. SPring-8 (Japan)

#### 3.1.1. Experimental Setup

**Figure 2:**
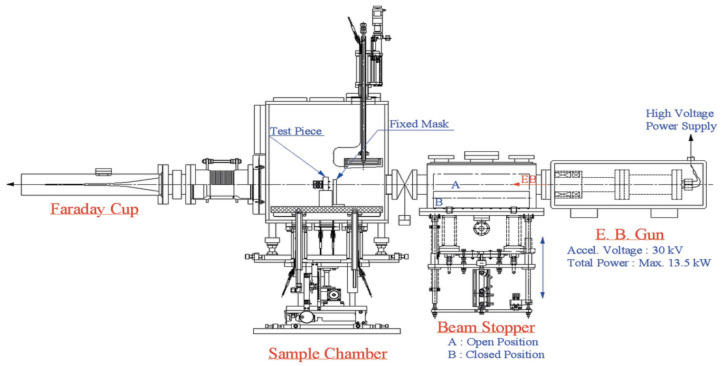
General diagram of the experimental setup [9].

The experimental setup is shown in Figure 2.

It mainly consists of an electron beam gun, a beam stopper, a sample chamber, and a Faraday cup. The main functions of different parts are given in Table 1.

**Figure 3:**
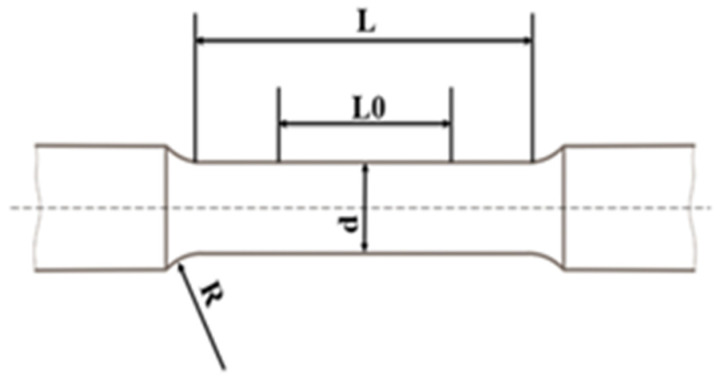
Schematic diagram of the test piece [15].

The experiments are performed according to the Standard JIS Z2279-1992 [15]. Additionally, the size of each test piece is shown in Figure 3.

(5) $$L_{0}/d\geq 1$$

(6) R/d≥2

(7) $$L\geq l_{0}+d\;,\;d=6,8,10\;or\;12\;\mathrm{mm}$$

**Figure 4:**
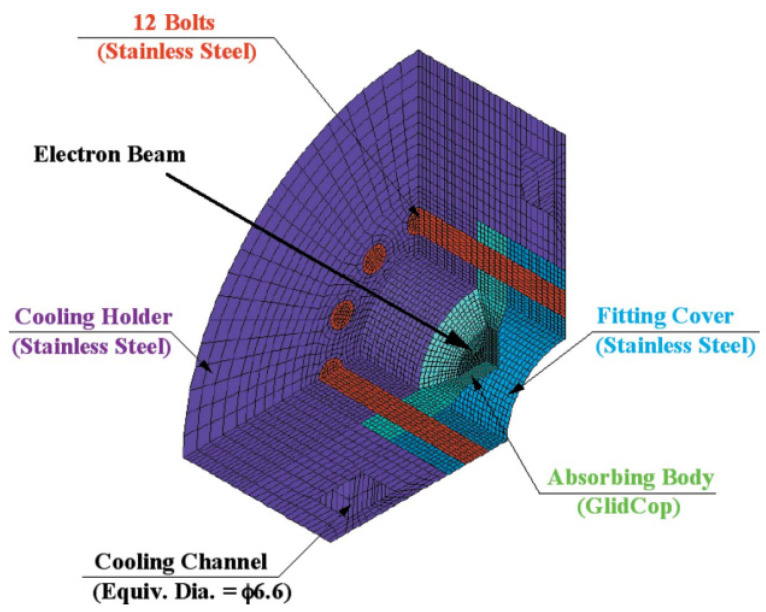
A quarter model of the specimen clamping assembly [9].

Figure 4 shows a quarter model of the specimen clamping assembly. The model consists of an absorber body made of GlidCop^®^ AL-15, an accessory cover made of stainless steel and a cooling bracket held in place by 12 bolts. The important feature of the assembly is its tapered configuration, which is designed to concentrate the strain in a local central area. Another feature is the tight retention of the binding force at the periphery of the GlidCop body using the axial tension provided by tightening the bolts [9].

In the experiment, an electron beam accelerated to 30 keV is guided onto the absorber after passing through a fixed mask made of tungsten with an 8 mm aperture. The beam stopper is driven by a pneumatic actuator to intercept the electron beam completely, thus generating a cyclic thermal load. One cycle consists of a seven min thermal loading condition and a five min unloading condition. This experiment consists of three cases, where the electron beam current is adjusted to correspond to 550 W, 600 W, and 650 W. The actual absorption is calculated from the flow rate and temperature difference between the cooling water inlet and outlet [9].

#### 3.1.2. Δ*ε_t_*-*N_f_* Curve

**Figure 5:**
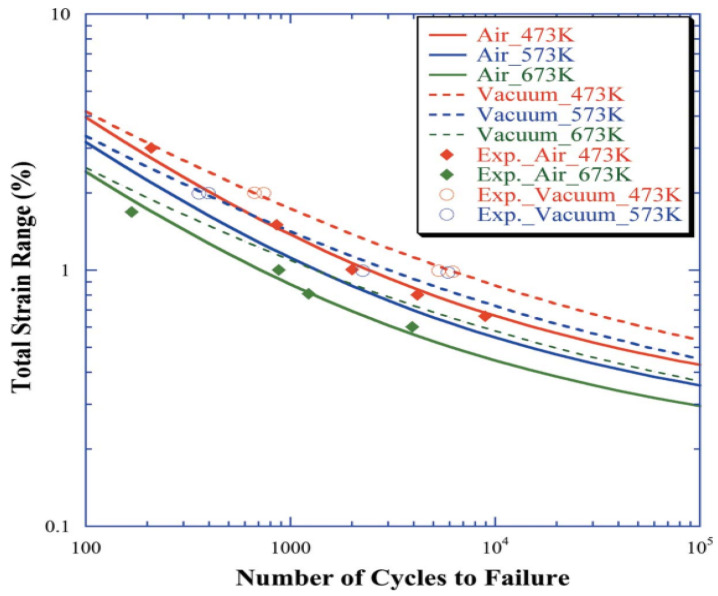
The *∆ε_t_*-*N_f_* plot depicts the change in strain on top of the specimen as the number of thermal cycles increases [9].

Figure 5 shows the relationship between the total strain range (Δ*ε_t_*) and the number of cycles to failure (*N_f_*) (the solid line indicates air, and the dashed line indicates vacuum), including the experimental results (markers). Predictably, the greater the total strain, the shorter the fatigue life. In addition, the fatigue life in a vacuum is longer than that in air. Furthermore, the environmental medium can seriously affect the creep–fatigue crack growth rate at high temperatures [16]. In addition, a study found that the oxide film around the cavity promotes the formation of fatigue cracks in the surface of a component, the minimum creep rate in air is greater than that in a vacuum, and the number of cracks is more than that in a vacuum [17]. Moreover, the air is more corrosive to the alloy than in a vacuum.

The total strain range (Δ*ε_t_*) is the sum of the plastic strain range (Δ*ε_p_*) and the elastic strain range (Δ*ε_e_*), which can be described by the approximate equations of the Manson–Coffin equation and the Basquin equation [18].

(8) $$\Delta \varepsilon_{t}=\Delta \varepsilon_{p}+\Delta \varepsilon_{e}=AN_{f}{}^{-\alpha }+BN_{f}{}^{-\beta }$$

where *A, B, α, β* are material properties and *N_f_* is the number of cycles to failure.

#### 3.1.3. Crack Length

After approximately each 30 “load-unloads”, the specimens are removed from the sample chamber and the surface is observed using a digital microscope. Once cracks were detected, they were observed using a field emission scanning electron microscope.

**Figure 6:**
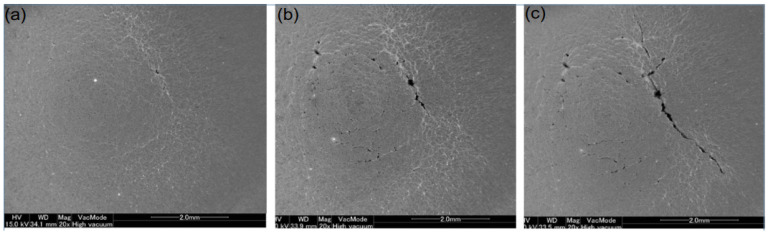
FE-SEM micrographs of the central region of the specimen after (**a**) 150 cycles, (**b**) 230 cycles, and (**c**) 260 cycles [9].

Figure 6a–c show FE-SEM photographs of the area around the center of the specimen using an electron microscope magnification of 20× when 150, 230, and 260 cycles were performed at an absorbed power of 650 W, respectively.

As indicated in the figures above, the first crack of about 0.22 mm in length was detected when the thermal cycling was carried out to 150 cycles, and when it was carried out to 230 cycles, the crack grew to 1.5 mm but showed a continuous hole-like pattern. A total of 260 cycles later, the crack length of about 3.5 mm was visible, and the metal can be identified as fatigue fracture.

**Figure 7:**
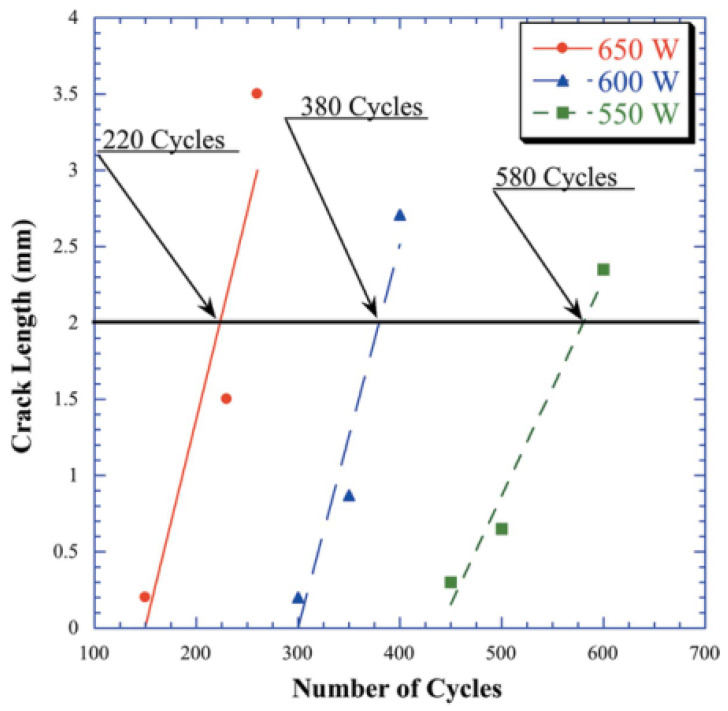
Crack length versus the number of cycles when the absorbed power is 550 W, 600 W, and 650 W [9].

Figure 7 represents the relationship between crack length and the number of cycles for all cases. Although it is not immediately clear that crack sprouting is equivalent to component fracture, crack extension is a precursor to fracture failure of the material. Therefore, based on the results of the low cycle fatigue test, the observed fracture life is considered as the number of cycles for which the maximum crack length reaches 2 mm.

Furthermore, when the point of crack length reaches 2 mm, the corresponding number of cycles decreases by about 34% when the absorbed power increases from 550 W to 600 W and about 42% when it increases from 600 W to 650 W. Additionally, the slope of curves indicates the crack sprouting speed at different temperatures. It can be found that the cracking propagation speed is faster at higher temperatures. Therefore, the higher the absorbed power is, the less the number of cycles required for the crack length to reach 2 mm, i.e., the fatigue life is shorter.

### 3.2. APS (USA)

#### 3.2.1. Test Setup

**Figure 8:**
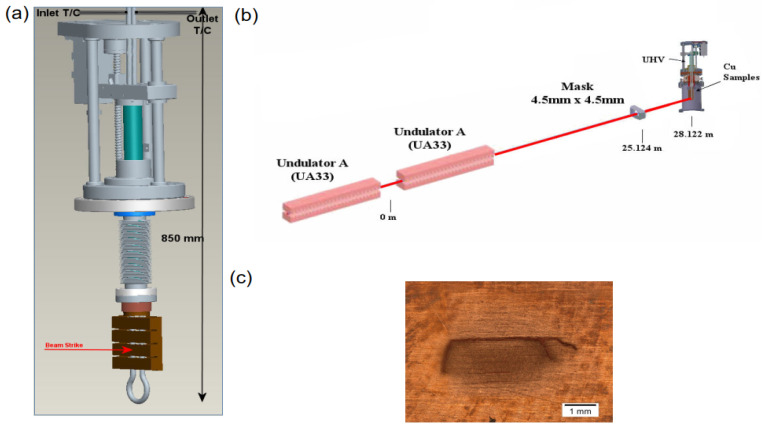
(**a**) Four GlidCop^®^ AL-15 samples mounted on a sample holder inserted into a vacuum chamber; (**b**) graph of a test sample with an X-ray beam from the test chamber and an X-ray beam from two fluctuators hitting the sample holder; (**c**) fatigue sample after 10,000 cycles at an absorbed X-ray power of 1185 W in a 1.2 mm × 4.3 mm area with multiple cracks [19,20].

A total of 45 rectangular GlidCop^®^ AL-15 samples (100 × 25 × 22 mm^3^) were prepared and welded to the cooling tube. Four samples were tested at each test (Figure 8a). Each sample was X-rayed (loaded-unloaded) 10,000 times at the area of 4.5 mm × 4.5 mm. The beam was turned on for 1 s and off for 9 s by using an upstream shutter. The beam came from two undulators (Figure 8b), whose gaps were varied to provide different thermal loads ranging from 674 W to 2250 W. The estimated maximum temperature range was 325 °C to 1096 °C for different samples. Examination of the samples showed different responses for different power loads, including initial cracking, multiple surface cracking, deep cracking, “spalling”, ablation, surface extrusion, and melting. The crack size and depth (deep crack state) in the specimens provided the necessary data for the thermal fatigue prediction model (Figure 8c).

#### 3.2.2. Equivalent Stress-Equivalent Total Strain Hysteresis Curves

To determine the total strain range and peak surface temperature for each specimen, a transient nonlinear finite element analysis was performed for each thermal loading condition. The average temperature and total strain range were used to predict the number of cycles to failure for each sample.

**Figure 9:**
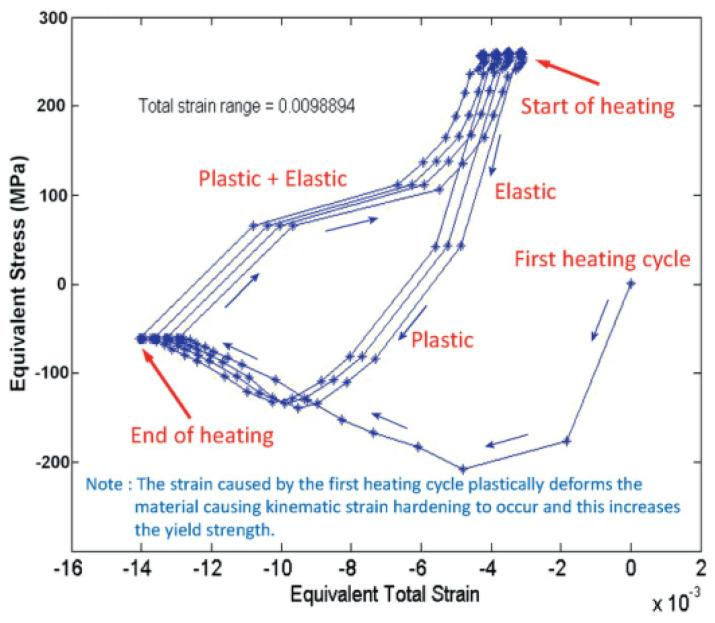
Typical equivalent stress-equivalent total strain hysteresis curves for specimens [19].

A multi-linear kinematic hardening model was also used, then the typical equivalent stress-equivalent total strain hysteresis curves were obtained, as shown in Figure 9.

It can be found that the strain generated in the first heating cycle causes plastic deformation if the yield point is exceeded, leading to the kinematic strain hardening, which locally increases the yield strength of the material [19]. Strain hardening is caused by crystal defects in the material, which can be grain boundaries, grain boundaries, cracks, or defects. When a material is subjected to an external force, the crystal defects deform, the grain is elongated along the direction of maximum deformation, and the lattice is distorted, thus improving the deformation resistance of the material. It can be seen from the resulting analysis diagram that only four loading cycles are needed for the total strain range to converge to a fixed value.

#### 3.2.3. Damage Morphology

**Figure 10:**
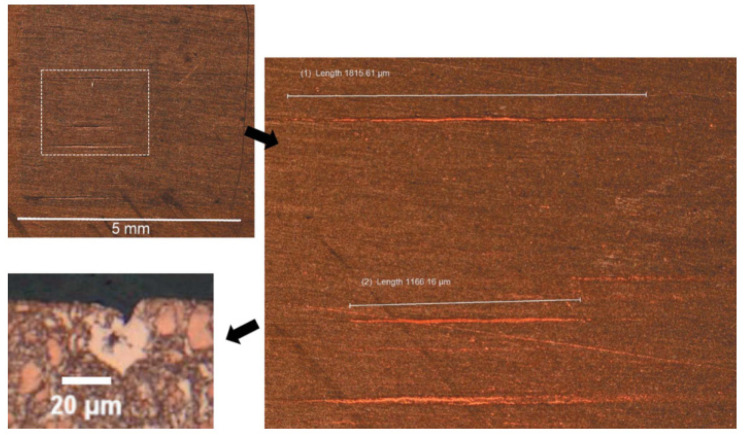
The “cat scratch” is a shallow area of surface grain shedding that is the result of surface thermal compression ejecting the remnants of weakly bound copper grains. The photograph in the upper left corner illustrates the visual appearance under normal lighting conditions and the origin of the name “cat scratch”. The image on the right shows a magnified view of the bright field. The lower left image shows a highly magnified cross-section through the 1.8 mm long scratch above [19].

It indicates that the “cat scratches” can be found in the samples, which may be small shallow cracks less than 2 mm in surface length. The “cat scratches” observed by metallurgical surface imaging are shown in Figure 10. It shows that the multiple parallel linear “cat scratches” can be observed on several GlidCop^®^ AL-15 test samples at higher thermal load or longer time. In addition, this type of crack will become the dominant failure mode and will be further propagated and leading to the ultimate fatigue failure.

**Figure 11:**
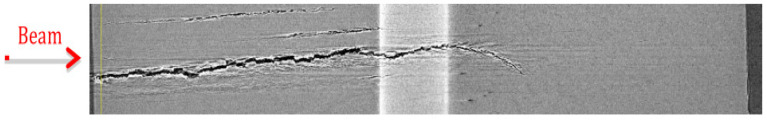
The micro-level cracks were measured with the nondestructive measurement method [20].

Moreover, the micro-level cracks can be measured with the nondestructive measurement method by using the high-energy X-ray. As shown in Figure 11, this method provides a closer view of the features around the main crack. It appears to be that the internal crack propagation path and beam are generally in the same direction. In addition, the localization and analysis of internal cracks can be achieved without damaging the sample after using this method.

## 4. Methods for Improving the Thermal Fatigue Resistance of Materials

Thermal fatigue is the fatigue failure phenomenon caused by the thermal stress (or thermal strain) cycle caused by the temperature gradient cycle. Therefore, the thermal fatigue resistance of materials can be improved by the following methods.

### 4.1. Structure Optimization

The optimization of the cooling structure includes many methods, such as reducing the slope of the beam receiving surface (which needs to consider the limitation of the installation space) and modifying the smooth beam receiving surface to the concave and convex block structure. The principle of the above-mentioned methods is to effectively avoid the high energy beam directly acting on the beam receiving surface, and the bumpy block structure also serves to disperse the stress and strain. The high concentrated stress at the corner can be reduced by designing the arc of the window on the surface of the absorber element [21,22].

APS incorporates vertical fins on the absorber surface to divide the beam footprint into two parts. The first part is intercepted by the fins, while the second part is intercepted by the grooves between the fins. At an incidence angle of 11°, the intercept distance between the two beams is 8 mm, while the water channel is only about 5 mm from the surface. Inner fins are also used in the water channel of the absorber to enhance heat transfer. The principle of this method is to increase the light contact area to avoid excessive temperature in a certain area while increasing the cooling contact area to remove heat faster and lower the temperature [23].

The crotch absorber of the Spanish synchrotron light source (ALBA) consists of two jaws. In addition, according to the applicable performance is divided into two; one is suitable for medium power size. The width of the teeth on this absorber is 10 mm, and the inclination of each tooth (concerning the radiation plane) is 8.8°, which reduces the normal incident power density by about 85%. The other one is the main (critical) absorber; this type of absorber will absorb radiation from the dipole installed inside it or behind it (near the radiation source point), where the power is the highest relative to the other types. To reduce the total strain and increase the number of cycles, this absorber has a tooth thickness of 6 mm, a tooth inclination of 6.6° and eight pin holes introduced in the absorber. Radii (rounded corners) were introduced to reduce stress and strain concentrations at the edges of the tooth tips. In addition, the ends of the pin holes were geometrically optimized to improve the heat transfer efficiency at this location [24]. The principle of this method is to reduce the concentrated thermal stress by digging grooves in the surface of the element to increase the light area.

To cope with the new radiation mode of the SPring-8 soft X-ray beamline station, a new metal mask is developed using volume heating technology. This technique involves brazing together beryllium and oxy-free copper (OFHC) using a metal surface heat load at a low z depth, such as beryllium or graphite. Thermal analysis simulations were performed after physical experiments were conducted to determine that the brazed joint had sufficient mechanical strength. The results showed that the ability of the matching metal material to absorb high thermal loads was improved by approximately 4 kW/0.2 m. The principle of this method is that OFHC has multiple cooling channels capable of absorbing and cooling the high-energy portion of the X-ray beam passing through the beryllium block and eliminating the heat load absorbed by the beryllium block through brief cooling. The thermal analysis results show that the heat absorbed by beryllium accounts for about 82.5% of the total heat absorbed by the element [25]. Otherwise, the structure has the advantage that the total required length in the beam axis direction is shorter than the past technology of the pre-slit.

### 4.2. Improvement of Film Coefficient

1. Increase the flow rate/velocity of cooling fluid [26].

For the convective heat transfer method, the key issue is to improve the cooling effect by increasing the film coefficient at the cooling inner wall. The film coefficient can be increased by appropriately increasing the fluid velocity and flow rate or reducing the diameter of the circular cooling tube. In addition, the physical properties of cooling fluid, such as density, specific heat, thermal conductivity, viscosity, etc., will also affect the heat transfer effect.

2. Insert the wire coil into the cooling tube.

There is currently an enhanced cooling method that is used to insert the wire coil into the cooling tube [27]. The enhanced cooling mechanisms are [28]:

(1) Increasing the cooling area
(2) Thinning or destruction of the boundary layer
(3) Promoting the formation of turbulence

In recent years, this method has also been applied and promoted at some synchrotron facilities. The wire coil is shaped similarly to a spring and is generally made of copper.

### 4.3. Selection of Materials

Copper metal materials with high heat conduction, high-temperature stability, and small photon absorption coefficient are usually selected to manufacture the high heat load components at the synchrotron front end. The total power of the first or second-generation synchrotron radiation devices is relatively low, and the heat load on the beam receiving surface of components cannot even cause the yielding of the materials (generally high conductivity oxygen-free copper, OFHC); therefore, no special means are needed to deal with the high heat load issue [29]. The third-generation synchrotron radiation light source has greatly improved the total power and peak power density, which can reach tens of kW and hundreds of W/mm^2^, respectively. Under this condition, if no corresponding measures are taken, the components are very vulnerable to thermal damage. According to the actual situation, considering the thermodynamic performance and cost of the specific material, for the aperture with low thermal power density, the applied material is generally oxygen-free high-conductivity copper (OFHC) or chromium zirconium copper (CuCrZr). Glidcop^®^ AL-15, with better mechanical properties, is generally used for fixed maskers and photon shutters, which bear a high-power density [21,30,31,32].

Specifically, the purity of copper in OFHC is greater than 99.95%. Copper has high thermal conductivity, good weldability, excellent plasticity and ductility, excellent cold workability, and non-magnetism, so the dispersed OFHC overcomes the shortcomings of low yield strength after annealing and poor creep resistance at high temperatures. Therefore, it has the characteristics of high-temperature resistance, high strength, and high thermal conductivity [33]. The chemical composition of chromium zirconium copper (CuCrZr) is (mass fraction %, Cu: 0.4–0.77, Cr: 0.15–0.35, Zr: 0.08–0.25). It has high strength, hardness and thermal conductivity, as well as good wear resistance. After the aging treatment, its hardness, strength, and thermal conductivity are significantly improved, and it is easy to weld [34]. Glidcop^®^Al-15 is an oxide dispersion-strengthened (ODS) copper that is widely used in high-heat load components around the world due to its high strength, especially at high temperatures.

Among the three materials, thermal conductivity and yield stress are two important indicators. The thermal conductivity of OFHC is 391 W/(mK), 1.18 times that of Glidcop^®^ AL-15, and CuCrZr, respectively. Additionally, the yield stress of Glidcop^®^ AL-15 is 420 MPa, 1.24 times and 1.2 times higher than that of OFHC and CuCrZr, respectively. Of course, the commercial costs of the applied materials should also be considered. Among the three materials, OFHC has the lowest price, about 70 RMB/kg, followed by CuCrZr, and the price of Glidcop^®^ AL-15 is the highest, which is more than ten times that of OFHC [26].

From the above content, we can conclude that when the thermodynamic performance parameters of OFHC can fully meet the actual working conditions, we choose the most economical OFHC. Although Glidcop^®^ AL-15 has a slightly lower thermal conductivity than OFHC, it has higher strength at higher temperatures; when the temperature and thermal stress under working conditions are too high, the priority principle is Glidcop^®^ AL-15. CuCrZr is not generally used unless specifically required.

It is worth pointing out that in addition to the selection of materials with good performance, special alloying elements can also be added to metal materials or non-metallic particles (nitride, oxide, etc.) as additives to achieve the purpose of improving its heat-resistant fatigue performance. For example, CuCrZrAg with stronger thermal fatigue resistance was formed by adding a trace silver element to CuCrZr alloy. The principle is that the Ag element can promote the formation of dispersed nanoparticles, thus improving the grain size and distribution of the alloy. These nanoparticles prevent the movement and diffusion of grain boundaries, thus improving the strength and stability of the alloy. The addition of silver elements can also improve the thermal conductivity, which is conducive to improving the thermal fatigue resistance of the alloy [35,36].

In addition, strengthening copper by mechanical alloying with some granular materials, such as silicate glass particles [37], silicon carbide [38], alumina [39] or nano-diamond [40], can keep the copper matrix stable at high temperatures and improve the thermal fatigue resistance.

### 4.4. Process Design

#### 4.4.1. Thermal Barrier Coating

Thermal barrier coating refers to a thermal protection technology that covers ceramic materials with high heat resistance, corrosion resistance, high thermal expansion coefficient, and low thermal conductivity on the surface of hot-end components in the form of coating, which can effectively reduce the temperature of metal components and, thus, extend the service life of hot-end components. It is widely used in gas turbines, aerospace engines, etc. [41,42]. A typical thermal barrier coating consists of a ceramic surface and a metal bonding layer. The main function of the ceramic surface layer is to isolate heat flow and corrosive media; The main function of the bonding layer is to alleviate the mismatch of thermal expansion between the ceramic layer and the metal substrate and prevent the metal substrate from oxidation at high temperatures [43,44,45].

A total of 6–8% Y2O3•ZrO2 is currently the most successful and widely used ceramic material for thermal barrier coating. The Thermodynamic properties of this material are shown in Table 2 [46].

Under the conditions of heating time and cooling time of 120 s and 300 s, the highest temperature of the experiment is 1323 K, the lowest temperature is 293 K, and the thermal fatigue test of gas cooling, the experiments of no pre-oxidation without high-temperature holding, no pre-oxidation with high-temperature holding, and pre-oxidation without high-temperature holding are carried out, respectively.

The experiment result shows that the fatigue life of the coating is the longest under the condition of no pre-oxidation and high-temperature maintenance. The fatigue life of the coating is the shortest when the coating is pre-oxidized for a long time without high temperature. For coatings, the typical failure modes are coating, spalling, and cracking, while oxidation usually occurs in the bond layer, which leads to oxidation playing an important role in coating failure [47]. In addition, thermal fatigue is easy to occur under the action of heat shock, which leads to the decrease of mechanical properties, which is also the main reason for the decrease in fatigue life.

The advantage of thermal barrier coating is that it is cheaper and faster to research than developing materials with better heat resistance. However, the thermal barrier coating is still limited in temperature; for example, 6–8% Y_2_O_3_•ZrO_2_ will undergo phase transformation and sintering after the temperature exceeds 1473 K, causing cracks or leading to coating failure. Therefore, more work needs to be conducted in the future.

#### 4.4.2. Strengthening Process

Shot peening is also a common process method to improve the fatigue resistance of materials. The mechanism of this process is to spray high-speed shot flow onto the surface of parts to make them undergo plastic deformation and form a strengthened layer of a certain thickness. Residual compressive stress will be generated on the surface of treated materials, which can increase the closing force of microcracks and block crack growth, thus prolonging the fatigue life [48]. Tan et al. [49] proposed a laser shock treatment method for the surface of this reinforced material. The participating compressive stress generated by this method is more than ten times that of shot peening. The experimental results of Liu et al. [50] show that the residual compressive stress of the alloy material under this process is greatly increased, and the thermal fatigue life is increased by 120% compared with the as-cast state.

Deep cryogenic treatment (DCT) is to keep the material far below room temperature for a period and promote the microstructure transformation of the material through heat exchange and tissue reconstruction during the cooling process, to improve the mechanical properties of the material. As an effective supplement to conventional heat treatment, this method has the advantages of being clean, pollution-free, high efficiency, and economy [51]. Li et al. [52] found that DCT could refine grain and martensitic lath bundles, thus improving the mechanical properties of metal materials. Adem et al. [53] found that during the DCT, a large amount of residual austenite was transformed into martensite, and fine carbide precipitates out on the matrix, thus improving the mechanical properties and wear resistance of the material. Experiments by Zhang et al. [51] show that after DCT, many fine and dispersed carbide precipitates from metal materials, hindering dislocation movement and having a nailing effect on grain boundaries, delaying crack growth rate and surface hardness decline rate, thus improving the thermal fatigue performance of samples.

The thermal fatigue life of materials can be improved by using grain size control technology, such as high-pressure torsion [54] and other methods to control grain size and distribution. The principle of this method is that when the grain size is reduced, the dislocation density of the material increases, and the interaction between the stress field and dislocation at the grain boundary and within the grain is enhanced, thus increasing the strength and plasticity of the material. In addition, smaller grain sizes can limit grain boundary diffusion and reduce the deformation and damage of the material. Reducing grain size can also enhance the phase stability of the material, reduce the energy and diffusion of the grain boundary, and improve the thermal fatigue life of the material. After RK et al. [55] treated metal samples by high-pressure torsion (HTP), grain refinement is obvious, root mean square strain increases, and dynamic precipitation occurs. The thermal stability of the samples was significantly enhanced, the ultimate tensile strength was increased by 2.5 times, and the fatigue endurance limit was increased by 20%.

## 5. Discussion and Conclusions

The thermal fatigue issue of high heat load components at the synchrotron radiation front end should be considered, and it is of great practical significance to solve this problem. In this paper, the thermal fatigue theory, experiments, and optimization of thermal fatigue-resistance properties of copper metals commonly used in synchrotron front end are reviewed. In addition, representative research on thermal fatigue by well-known institutions, such as SPring-8 (Japan) and APS (United States), is also introduced. The conclusions are as follows:

(1) In the area of enhancing the thermal fatigue resistance of components through structural design, the previous, more conservative design guidelines for thermal stress are broken, and the design conditions for high thermal load components are redefined by extending from the previous elastic deformation region to the plastic deformation region.

(2) The thermal fatigue properties of applied materials can be tested via low-cycle thermal fatigue experiments, and the relationship between total strain and fatigue life can be quantified using the modified Manson–Coffin formula. In addition, the initiation and propagation of micro-crack are the main modes of thermal fatigue failure.

(3) The thermal fatigue resistance performance of high heat load components can be improved by optimizing the structures. For example, to adjust the cooling wall thickness, grazing incidence angle of the beam, cooling channel layout, etc. Furthermore, it can be realized by changing the shape of the beam receiving surface (such as changing the smooth surface into a concave, convex block structure), as well as improving the film coefficient at the cooling inner wall and selecting the better/more appropriate materials with better thermal performance.

(4) The samples were treated with high-energy X-ray beams for micro chromatography and diffraction experiments and then reconstructed by absorption X-ray chromatography. The nondestructive detection of microcracks was realized.

In addition, the methods of improving the thermal fatigue performance of metal materials are also proposed. For example, the structural design increases the heating area and reduces the concentrated stress. Alloys with strong thermal fatigue resistance can be obtained by doping metal or non-metal particles into existing metal materials. Thermal barrier coating, laser shock, cryogenic treatment, and other new thermal processes can effectively reduce the thermal fatigue of metal materials and improve fatigue life. The thermodynamic properties of OFHC, CuCrZr, and Glidcop^®^AL-15 materials and their selection suggestions in terms of economic feasibility are described. We believe that the innovative heat treatment processes and surface treatments can effectively improve the thermal fatigue properties of materials, and a lot of work is needed in this field in the future.

## Figures and Tables

**Table 1:** Experimental components and their functions.

Part	Function
Electron beam gun	Generating, accelerating, and converging the high-energy-density electron beam, used as the heat source
Beam stopper	Driven by a pneumatic actuator to intercept the electron beam completely
Sample chamber	Placement of test samples
Faraday Cup	To measure the incident intensity of charged particles. The measured current can be used to determine the number of incident electrons or ions

**Table 2:** Thermodynamic properties of 6–8% Y_2_O_3_•ZrO_2_.

Material	Melting Point (°C)	Thermal Expansivity (K^−1^)	Density (g/cm^3^)	Elasticity Modulus (GPa)	Thermal Conductivity W/(m·k)
6–8% Y_2_O_3_•ZrO_2_	2700	10.7 × 10^−6^	6.4	50	2.5–4.0

## Data Availability

Not applicable.
